# Electrochemical Sensors Based on Covalent Organic Frameworks: A Critical Review

**DOI:** 10.3389/fchem.2020.601044

**Published:** 2020-11-26

**Authors:** Sidi Chen, Baiqing Yuan, Gang Liu, Daojun Zhang

**Affiliations:** ^1^School of Chemistry and Materials Science, Ludong University, Yantai, China; ^2^Henan Province Key Laboratory of New Optoelectronic Functional Materials, College of Chemistry and Chemical Engineering, Anyang Normal University, Anyang, China

**Keywords:** electrochemical sensor, covalent organic frameworks (COFs), electrical conductivity, nanoscale COFs, conductive substrate, metal-covalent organic frameworks (MCOFs)

## Abstract

The metal-free cousins of metal-organic frameworks, covalent organic frameworks (COFs), are a class of pre-designable crystalline polymers composed of light elements and connected by strong covalent bonds. COFs are being given more and more attention in the electrochemical sensor field due to their fascinating properties, such as highly tunable porosity, intrinsic chemical and thermal stability, structural diversity, large specific surface area, and unique adsorption characteristics. However, there are still some key issues regarding COFs that need to be urgently resolved before they can be effectively applied in electrochemical sensing. In this review, we summarized recent achievements in developing novel electrochemical sensors based on COFs, and discussed the key fundamental and challenging issues that need to be addressed, including the mechanisms underlying charge transport, methods to improve electrical conductivity, immobilization methods on different substrates, synthesis strategies for nanoscale COFs, and the application of COFs in different fields. Finally, the challenges and outlooks in this promising field are tentatively proposed.

## Introduction

Covalent organic frameworks (COFs), a new class of multifunctional porous crystalline materials, are two- or three-dimensional (2D or 3D) porous crystalline materials built by light elements (C, B, O, Si, and N) via strong covalent bonds (C-N, C=N, C=C-N, B-O) (Xue et al., 2017; Chen et al., 2019; Wang and Zhuang, 2019; Zhu et al., 2019). Since the first report in 2005 by Cote et al. (2005), COFs have attracted more and more attention due to their fascinating properties, and many novel COFs have been synthesized (Waller et al., 2015; Lohse and Bein, 2018). Compared with other materials, COFs have many unique properties, such as highly tunable porosity, large specific surface area, unique adsorption characteristics, ordered channel structure, and intrinsic chemical and thermal stability, which make them outstanding in many fields including separation, gas adsorption, analysis, energy conversion and storage, and electrochemical sensing (Wu and Yang, 2017; Wang J. et al., 2018; Zheng et al., 2019). Ordered network configuration and multiple active acupoints give COFs a large adhesion surface, which is superior to 2D graphene nanosheets. In comparison with another class of porous crystalline material MOFs, COFs have thermal and chemical stabilities due to the involvement of covalent bonds (Li et al., 2020b; Yusran et al., 2020b).

To ensure the growth of the crystalline structures, the chemical reactions involved in the construction of COFs need to be reversible to render the self-healing ability to repair structural defects caused by mismatched covalent linkages. Up to now, researchers have proposed six synthetic methods for COFs, which refers to solvothermal, ionothermal, room-temperature, mechanochemical synthesis, interfacial synthesis, and microwave synthesis (Geng et al., 2020). Solvothermal synthesis is one of the most common methods to fabricate COFs, occurring in a sealed system at a specific temperature and pressure (Chen et al., 2019). For example, BND-TFB COFs were synthesized through the solvothermal method with improved material quality and a shorter reaction time. Ordered and amorphous microporous polytriazine networks were prepared through ionothermal synthesis by the trimerization of nitriles in a ZnCl_2_ melt at 400°C (Vitaku and Dichtel, 2017). The material exhibited a very large surface area that can be used for gas storage, sensors, or catalyst carriers (Kuhn et al., 2008). TpBD-based COFs were prepared via room-temperature synthesis, which is an attractive way to construct COFs for the case of fragile organic units or sensitive substrates (Yang et al., 2015). Mechanochemical synthesis is a simple, economical, and green method in which building blocks are mixed in a mortar and ground under ambient conditions to yield the COFs (Geng et al., 2020). The interfacial synthetic strategy is a novel and efficient method for fabricating COF thin films with controllable thickness. For the very first time, mesoscale covalent self-assembly was explored to fabricate self-standing crystalline porous thin films without defects at the liquid-liquid (DCM-water bilayer) interface (Sasmal et al., 2019). Microwave synthesis is a simple and efficient approach to building COFs. The melamine-based porous polymeric network SNW-1 was synthesized by a microwave-assisted synthesis route (Zhang et al., 2012).

**Figure 1:**
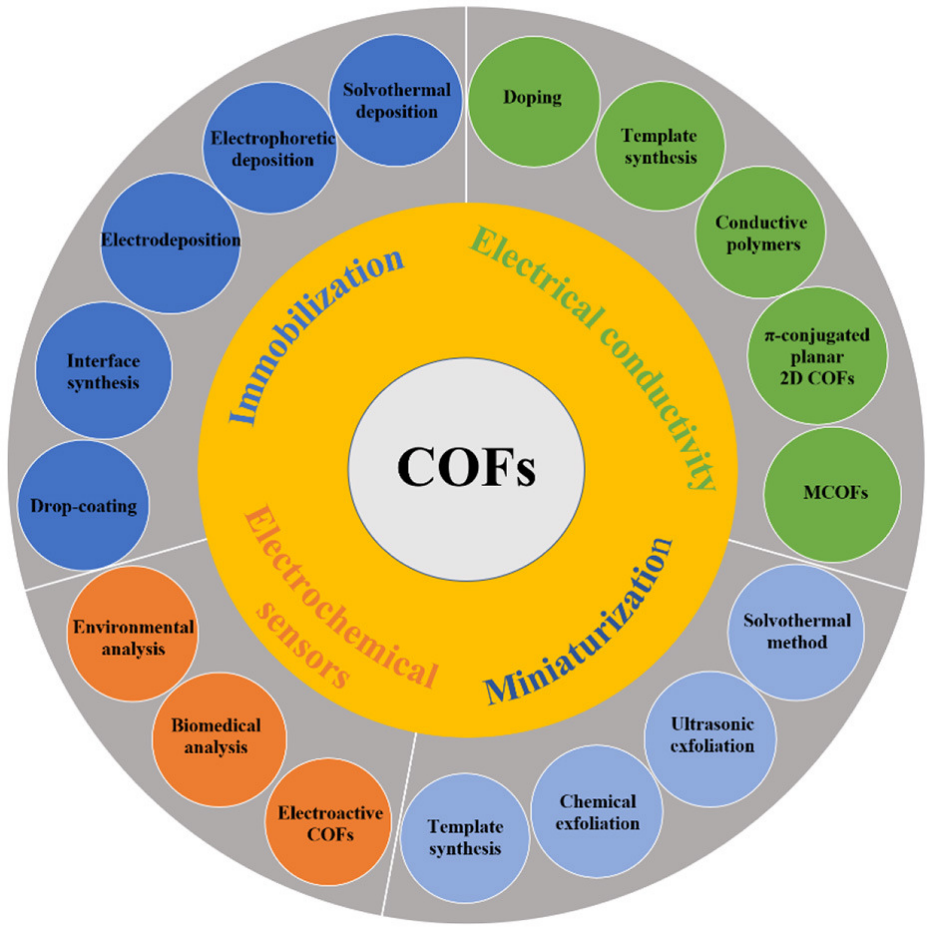
Schematic illustration of COF-based electrochemical sensing.

Electrochemical sensors work by reacting with analytes to produce electrical signals which are proportional to its concentration. A typical electrochemical sensor consists of a sensing electrode (or working electrode) and a counter electrode separated by a thin electrolytic layer (Karimi-Maleh et al., 2019). Recently, electrochemical sensing has gained extensive attention in multiple fields, such as pharmacy, clinical diagnosis, environmental monitoring, and food safety, because of its low cost, sensitive response, and simple operation (Yan et al., 2019; Yang et al., 2019; Liang et al., 2020). COFs have been widely exploited in electrochemical sensing due to their unique properties (Liang et al., 2019a; Sun et al., 2019a), which can improve the sensitivity of electrochemical sensors. For example, COFs possess a highly ordered porous structure, functional groups, and available holes, providing a large active surface in which to load electroactive molecules. In addition, their better biocompatibility also improves the stability of the electrochemical sensor (Ding et al., 2014; Li et al., 2020a). However, some key issues regarding COFs need to be urgently resolved before they can be effectively applied in electrochemical sensing. Herein, we present a critical review on the recent advances of COFs and their application in electrochemical sensors, with focus on the mechanism and method/strategy for improving electrical conductivity, the immobilization on different substrates, miniaturization, and application in electrochemical sensors (Figure 1). The challenges and outlooks toward COF-based electrochemical sensing are also discussed. We hope that the review will guide readers to design and develop COF-based materials for electrochemical sensing applications.

## Improving the Electrical Conductivity of the COFs

**Figure 2:**
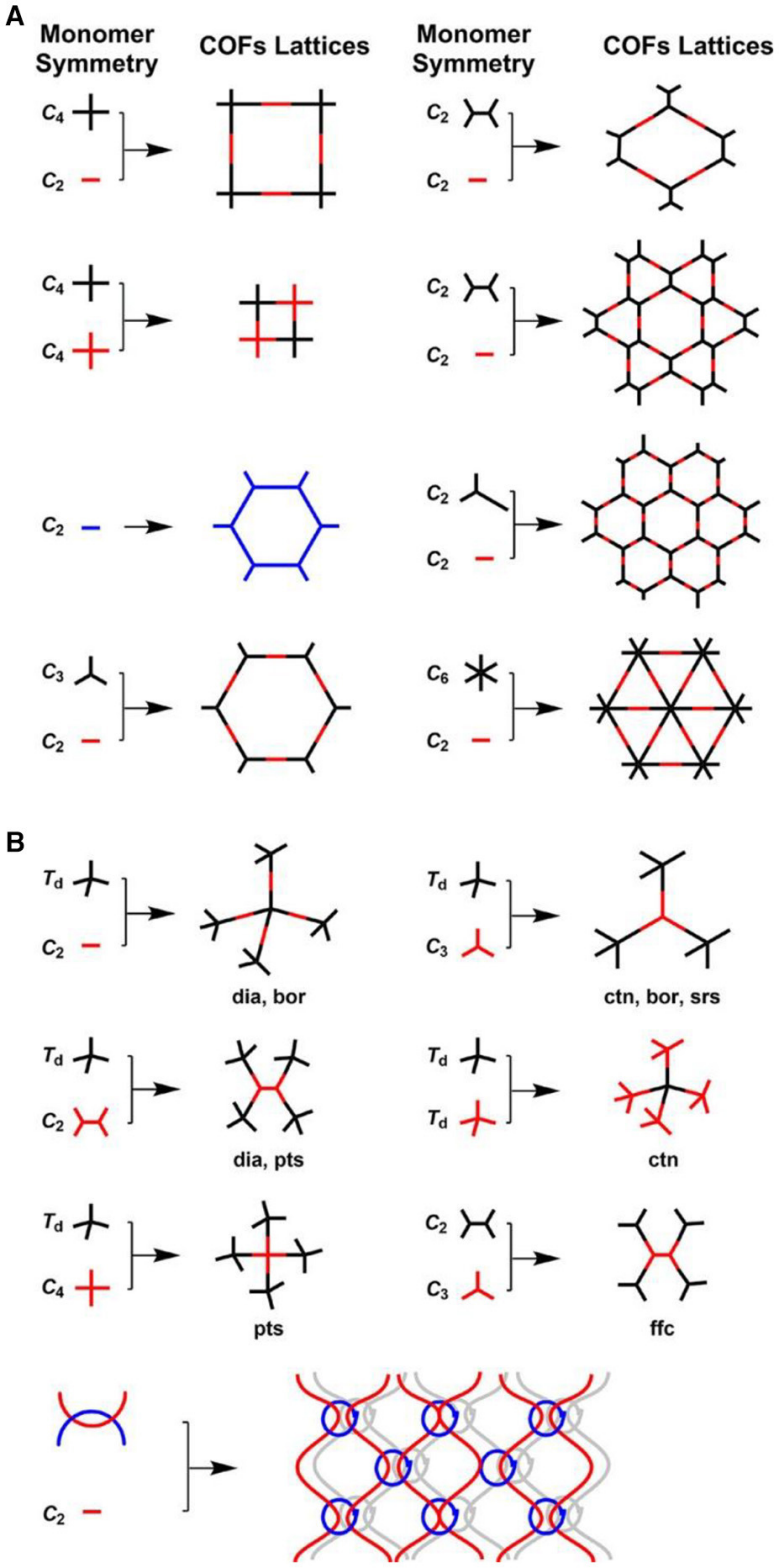
Basic topological diagrams for the design of 2D and 3D COFs. Reproduced with permission from Geng et al. (2020). Copyright 2020, American Chemical Society.

A topology design diagram can be used to guide the synthesis of 2D or 3D COFs in which the geometry of the selected organic monomers **determine** the primary-order structure **in either a 2D or** 3D manner (Geng et al., 2020), as shown in Figure 2. In 2D COFs, planar building blocks are covalently connected in the lateral crystallographic direction and further stacked together in the vertical direction by van der Waals. By contrast, the design of 3D COFs requires at least one building block to possess Td or orthogonal geometry that controls the development of the skeletons into a 3D structure. To ensure the growth of the crystalline structures, the chemical reactions involved in the formation of COFs need a certain reversibility which creates the self-healing ability to repair structural defects (Huang et al., 2016). However, the self-healing process is not sufficient, resulting in abundant defects in COFs. In addition, the low molecular conjugation of π-electrons cause electron localization. The issues lead to the intrinsic poor conductivity of bulk COFs, which limits their application in electrochemical sensing.

**Figure 3:**
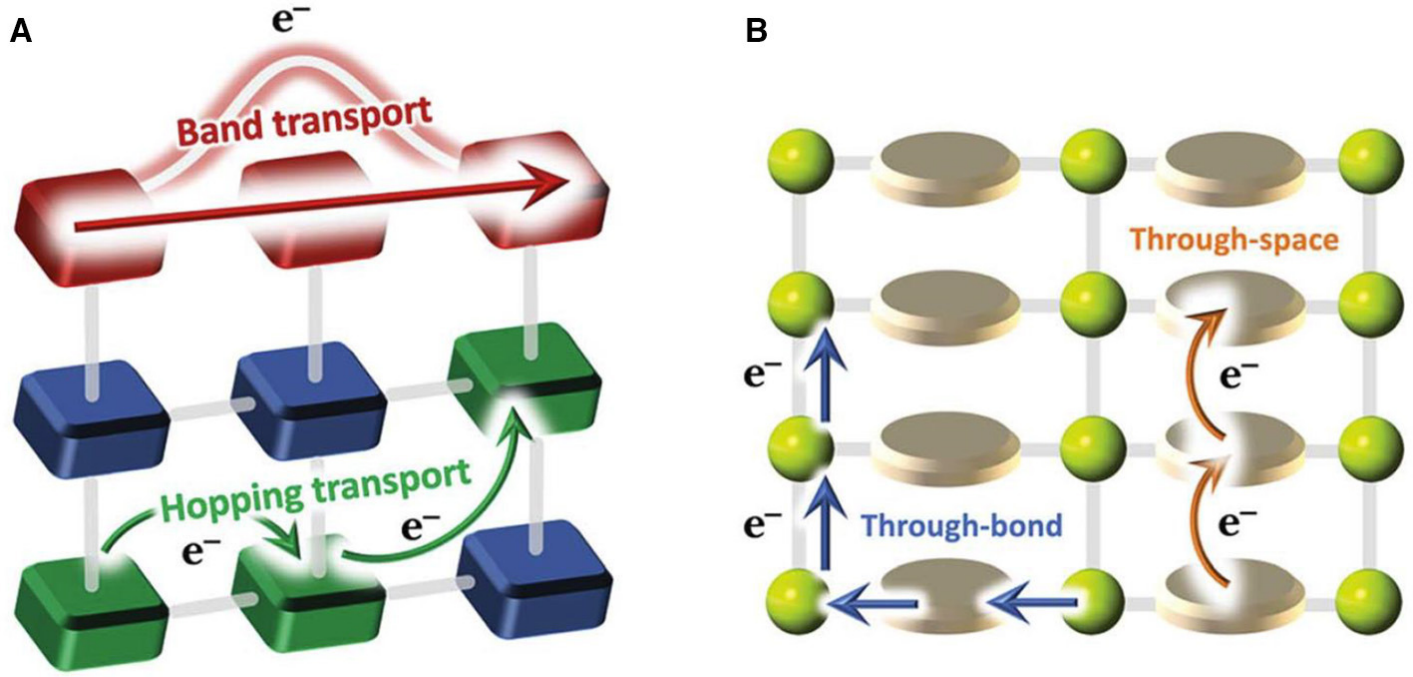
Schematic diagrams of charge transport modes (band transport and hopping transport) **(A)** and pathways **(B)**. Reproduced with permission from Calbo et al. (2019). Copyright 2019, The Royal Society of Chemistry.

There are two mechanisms for charge transport in COFs: hopping transport and ballistic (or band-like) transport (Figure 3) (Xie et al., 2020). In hopping mode, the charge carriers (electrons/holes) hop between isolated, non-bonded neighbor sites (donor and acceptor sites), where the charge carriers are localized. As for band transport, the charge carriers are delocalized and continuous energy bands are formed. Conductive COFs can be therefore categorized into two categories: through space and through bonds (Meng et al., 2019). Instead of a single bond connection including borate and imine linkage, full annulation of building blocks through aromatic linkages can promote efficient charge delocalization, suggesting a promising strategy for conjugated 2D structure generation to achieve a through-bond charge transport (Guo et al., 2013). Through-space charge transport relies on maximizing orbital overlap with lowered energy for charge transport through a strategic choice of building blocks. For example, π-stacking was explored to design COF-based materials with improved electrical conductivity (Wan et al., 2011). The principal strategy to guide the synthesis of conductive COFs is to obtain a highly conjugated and crystalline structure with few defects.

The low intrinsic conductivity of COFs still imposes a great challenge for their applications in electrochemical sensing (Meng et al., 2019; Wu et al., 2019; Xu L. et al., 2019). This problem could be overcome via the following methods and strategies including doping with oxidants and guest molecules, template synthesis, introducing conductive polymers, π-conjugated planar 2D structures, and the metalation of COFs.

The electrical conductivity of COFs can be improved by designing high-supply electronic blocks with electron acceptor dopants. For example, the electrical conductivity of COFs could be tuned by doping with iodine or TCNQ, resulting in high conductivity up to 0.28 S/m (Cai et al., 2014). In addition, this doping strategy is broad enough that it can be used to improve the electrical conductivity of many kinds of COFs.

Template synthesis is another powerful method to improve the conductivity of COFs on a conductive template surface. COFs with highly ordered pore channels (COF_TTA−DHTA_) were synthesized on an amino-functionalized carbon nanotube (NH_2_-f-MWCNT) from TTA and DHTA via imine linkages (Sun et al., 2017). The MWCNT@COF_TTA−DHTA_ not only had electrical conductivity but also possessed excellent crystallinity, regular pore channels, and a high surface area. The COF_BTA−DPPD_-rGO composite was also synthesized by this method at room temperature under ambient conditions (Xu L. et al., 2019). Coupling the improved conductivity from rGO, COF_BTA−DPPD_-rGO exhibited an enhanced electrochemical performance, which might be attributed to the synergistic effect of the π-conjugated COF_BTA−DPPD_ being fully covered on the conductive rGO surface.

Introducing a conductive polymer into the channel of COFs can also increase its conductivity. PEDOT is one of the most widely investigated conductive polymers because of its excellent electronic properties and high stability. One strategy includes electropolymerizing PEDOT into the pores of redox-active 2D COF films (Mulzer et al., 2016). PEDOT-modified COF films can accommodate high charging rates (10–1,600 C) without compromising performance and exhibit 10-fold current response relative to unmodified films and stable capacitances for at least 10,000 cycles. However, the disadvantages of electropolymerization and requirement of COF films as a precursor make it difficult to scale up to high-throughput production lines for practical application. Recently, a novel method to introduce PEDOT to improve the electrical conductivity of the COFs was reported by using an *in-situ* solid-state polymerization inside the nanochannels (Wu et al., 2019). The resulting PEDOT@AQ-COFs showed an electrical conductivity value of 11 0 S/cm at room temperature and a remarkably improved storage performance. This approach will serve as a promising strategy for increasing the electrical conductivity of COFs and extending the applications of COF materials.

The fourth method is the molecular design strategy focusing on planar 2D COFs, in which the formation of π-conjugated sheets can promote the delocalization of charge, has yielded metallic conductivities (Meng et al., 2019). A novel intrinsically conductive 2D COF was synthesized through the aromatic annulation of 2,3,9,10,16,17,23,24-octa-aminophthalocyanine nickel (II) and pyrene-4,5,9,10-tetraone. The intrinsic bulk conductivity of the COF material could be up to 0.0025 S/m, and increased by 3 orders of magnitude upon I_2_ doping. In addition, 3D electroactive TTF-based COFs were reported with high crystallinity and large permanent porosity, in which these TTF-based COFs were redox active to form organic salts that exhibit outstanding electric conductivity (Li et al., 2019).

In order to further enhance the electric conductivity, metal ions were introduced into the COFs lattice to form conductive metal-covalent organic frameworks (MCOFs), which can be synthesized through either direct synthesis or post-synthetic metalation by using planar and large π-electronic macrocycles as the building and paring units for the metal (Dong et al., 2020; Xie et al., 2020). Compared with metal free COFs, MCOFs not only have higher intrinsic conduction, but also exhibit superior electrocatalytic activity due to the presence of a metal component. 2D and 3D MCOFs have been prepared by using π-electron rich building blocks, such as porphyrin (Lin et al., 2015), phthalocyanine (Spitler et al., 2012), bipyridine (Aiyappa et al., 2016), and dehydrobenzoannulene (Baldwin et al., 2016).

## Immobilization of COFs on Different Substrates

It is an essential procedure to modify COFs on different electrodes for electrochemical sensing applications. The fabrication of ultrathin COF films is still very challenging, since the poor COF-substrate affinity hampers the nucleation of COF crystallites. Herein, immobilization methods and strategies for COFs on different substrates were summarized, such as solvothermal growth/deposition, electrophoretic deposition, electrochemical deposition, interfacial polymerization, and drop-coating, as shown in Table 1.

**Table 1:** Immobilization methods and strategies for COFs on different substrates.

**Substrates**	**COFs**	**Synthetic units**	**Immobilization methods**	**Thickness**	**References**
GCE	POR-COFs	TAPP	Electrochemical deposition	/	Tavakoli et al., 2019
ITO	COF-300	Tetrakis(4-aminophenyl) methane/terephthalaldehyde	Electrophoretic deposition	0.4–24 μm	Rotter et al., 2019
ITO	COF-5	Benzene-1,4-diboronic acid/2,3,6,7,10,11-hexahydroxytriphenylene hydrate	Electrophoretic deposition	0.4–24 μm	Rotter et al., 2019
ITO	BDT-ETTA COF	BDT/ETTA	Electrophoretic deposition	0.4–24 μm	Rotter et al., 2019
AAO	Imine-based COFs	1,3,5triformylphloroglucinol/p-phenylenediamine	Solvothermal growth	/	Shi et al., 2019
PSF	TpPa-COFs	1,3,5triformylphloroglucinol/p-phenylenediamine	Interfacial polymerization	0.29–1.12 μm	Wang R. et al., 2018
α-Al_2_O_3_	COF-320	tetra-(4-anilyl) methane and 4,4′-biphenyldicarboxaldehyde	Solvothermal growth	4 μm	Lu et al., 2015
GO	COF-1	1,4-benzenediboronic acid	Solvothermal growth	10–250 nm	Zhang X. et al., 2019
GCE	COF	BDBA/1,4-dioxane–mesitylene	Drop-coating	/	Zhang T. et al., 2019
Au electrode	DAAQ-TFP COF	DAAQ/TFP	Solvothermal growth	60–560 nm	DeBlase et al., 2015
ITO/FTO/platinum	2D COFs	DAB/TFP	Solvothermal growth	200 nm	Gou et al., 2016

**Figure 4:**
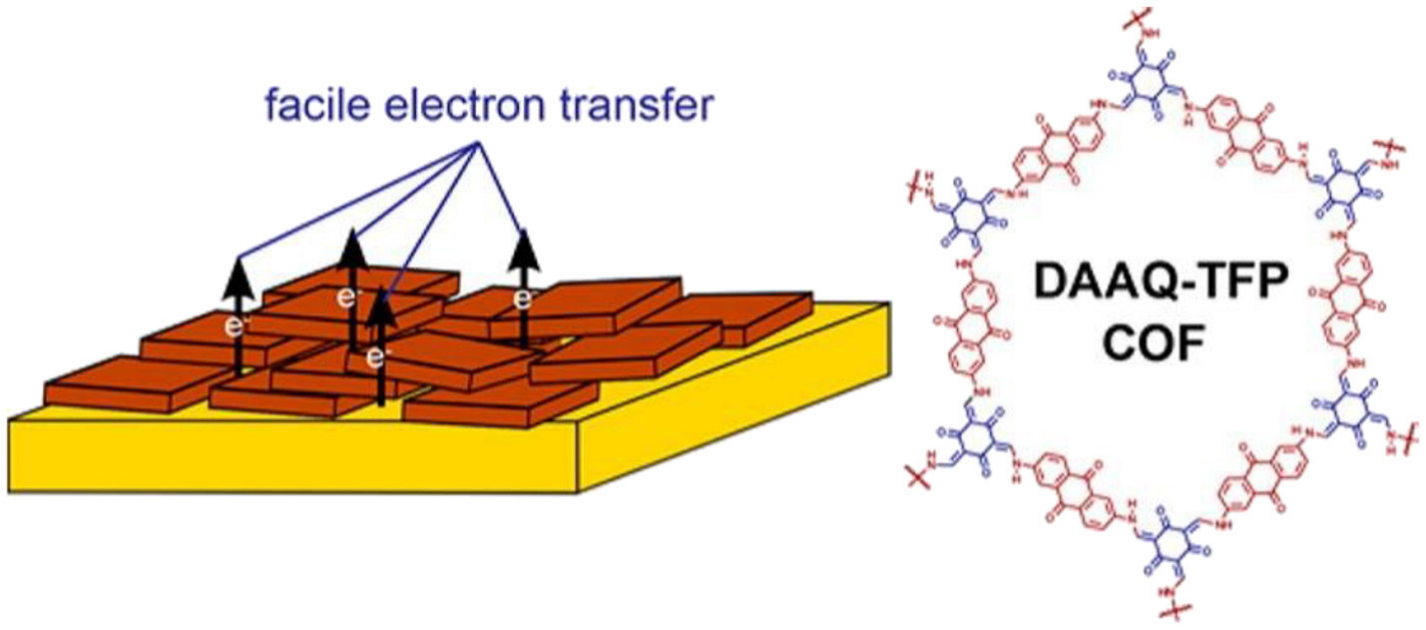
Oriented thin film of DAAQ-TFP COFs for efficient redox processes. Reproduced with permission from DeBlase et al. (2015). Copyright 2015, American Chemical Society.

Solvothermal growth/deposition is found to be an efficient method to immobilize DAB-TFP COF thin films on different substrates (indium tin oxide, fluorine doped tin oxide, and platinum substrates) (Gou et al., 2016). The oriented thin films of a redox-active 2D β-ketoenamine COF on Au was first fabricated by solvothermal growth, and the film thickness was controlled by varying the initial concentrations of the monomers (DeBlase et al., 2015). The oriented COF film modified electrode exhibit a 400% increase in capacitance scaled to the electrode area as compared to those functionalized with the randomly oriented COFs powder (Figure 4). Recently, COF-1 with an ordered channel structure and precise pore size was synthesized and attached onto the surface of graphene oxide (GO) by *in-situ* growth, which improves the dispersity and stability in water over COF-1 (Zhang X. et al., 2019).

The fabrication of COF film and coatings on conducting substrates was demonstrated in an electric field by electrophoretic deposition, which is suitable for depositing 2D and 3D COFs linked by imine or boronate ester bonds, such as BDT-ETTA COFs, COF-300, and COF-5 (Rotter et al., 2019). By controlling the key parameters including particle concentration, duration, and applied potential, deposition with precise thickness can be achieved. In addition, co-deposition of different COFs as well as COFs/Pt nanoparticles from mixed suspensions were also presented.

Solvent intractability and sluggish condensation kinetics have limited the synthesis and processing of 2D or 3D COFs. In order to resolve the problem, poly(5,10,15,20-tetrakis(4-aminophenyl)porphyrin)-COFs (POR-COFs) with a high crystalline order were electrochemically synthesized via the formation of phenazine linkages by controlling the temperature, potential scanning rate, and electrode materials and co-crystallization with pyridine (Tavakoli et al., 2019). The pyridine sublattice not only stabilized the Py-POR-COFs superlattice but also controlled the interlayer spacing and stacking in this class of materials, resulting in enhanced ORR activity.

A facile and simple strategy, interfacial polymerization, was developed for the direct synthesis of imine-typed COFs on polysulfone substrates to produce composite membranes (Wang R. et al., 2018). The prepared membranes exhibited superior long-term stability and stability even in highly acidic/basic conditions. The COFs/polysulfone composite membranes had the advantage of large-scale production, showing potential application for the treatment of wastewater and the removal of pharmaceutical wastes from water.

The synthesized COFs can also be dripped onto different electrodes and then dried at room temperature to achieve their modification. For example, COF nanosheets were modified on GCE by this method for signal amplification, which was applied for sensitive biomarker detection (Zhang X. et al., 2019).

## Nanoscale COFs

**Figure 5:**
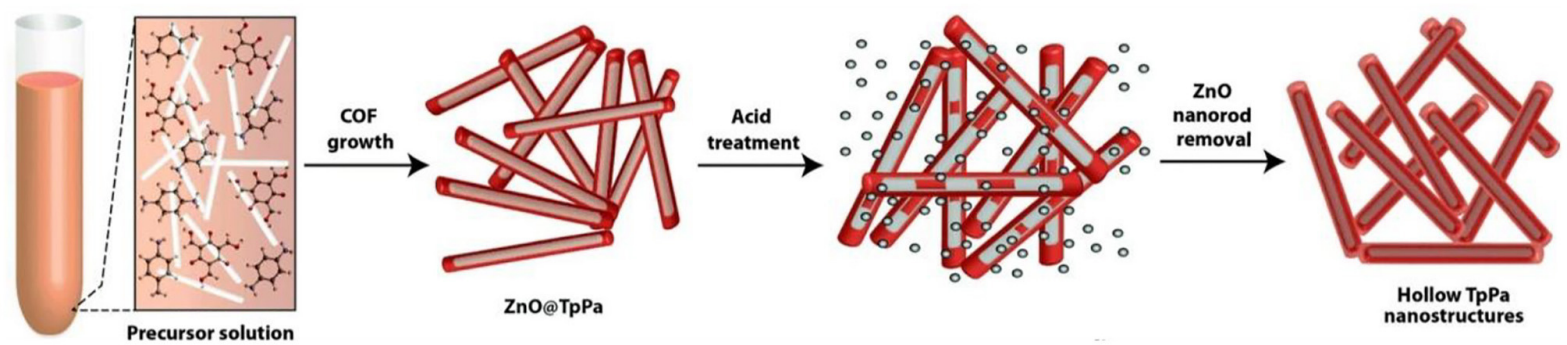
Synthesis of COF nanotubes via a template-mediated strategy. Reproduced with permission from Pachfule et al. (2015). Copyright 2015, The Royal Society of Chemistry.

Large size COFs lead to low active area, low mass transfer rate, and difficult modification as well as poor stability on the electrode, which will influence the stability, reproducibility/repeatability, and sensitivity. The miniaturization of COFs will solve this problem. The existing methods for nanoscale COF preparation includes the polymer-assisted solvothermal method, high-power ultrasonic exfoliation, steric hindrance-induced chemical exfoliation, and the template synthesis. However, these procedures are usually tedious and need strict synthetic conditions. Recently, a nanoscale COF prepared via a facile synthetic approach under ambient conditions was reported (Guan et al., 2019). Imine-linked TPB-DMTP-COFs was prepared through the reaction of 1,3,5-tris (4-aminophenyl) benzene and 2,5-dimethoxyterephthaldehyde under mild conditions (CH_3_CN, 25°C, 12 h) with the aid of acetic acid and polyvinylpyrrolidone (PVP). Unlike traditional solvothermal COFs synthesis, this approach does not need any vigorous reaction conditions, such as a solvothermal and inert atmosphere. More importantly, scaling up to a gram-scale nanoscale COF synthesis was easily achieved. In order to decrease the size of COFs, a template-mediated synthesis of hollow tubular COFs using a twostep strategy was reported by Pachfule et al. (2015). ZnO nanorods were wrapped with COF layers by a typical Schiff-base reaction of 1,3,5-triformylphloroglucinol and p-phenylenediamine. Then the inside templates were etched by acid to leave the hollow nanostructures in quantitative yield (Figure 5).

## Applications of COFs in Electrochemical Sensing

Based on their structural characteristics, COFs are endowed with unique properties, and have been successfully applied in electrochemical sensing. Nowadays, an electrochemical sensing platform based on COFs is widely used in the fields of environmental pollutant and biomedicine analysis. Herein, the applications of COFs in different fields are comprehensively summarized (Table 2). Furthermore, we focus the application of electroactive COFs in ratiometric electrochemical sensors which will be a promising field.

**Table 2:** The analytical performances of electrochemical sensors based on different COFs.

**Electrode**	**Analyte**	**Technique**	**Potential**	**Detection limit**	**Linear range**	**Sensitivity**	**References**
DQ-COF/Ni/ITO	Hydrazine	AP	0.8 V	0.07 μM	0.5–1,223 μM	1.81 μA cm^−2^μM^−1^	Liang et al., 2020
p-COF/AE	EGFR	DPV	0.2 V	5.64 × 10^−3^ pg mL^−1^	0.05–100 pg mL^−1^	/	Yan et al., 2019
3D-KSC/COF_TAPB−PDA_/PtNPs	H_2_O_2_	AP	0.04 V	0.006 μM	0.0185–5.4 μM 5.4–3055.4 μM	2.58 μA cm^−2^μM^−1^ 449.33 μA cm^−2^ mM^−1^	Yang et al., 2019
3D-KSC/COF_TAPB−PDA_/CuNPs	Glucose	AP	0.5 V	1.54 μM	4.69–1,570 μM 1,570–7,070 μM	2.128 mA cm^−2^ mM^−1^ 0.829 mA cm^−2^ mM^−1^	Yang et al., 2019
Au NPs/BPene/Fe_3_O_4_-COF/GCE	PSA	DPV	0.3 V	30 fg mL^−1^	0.0001–10 ng mL^−1^	/	Liang et al., 2019a
MIP/GO@COF/GCE	SDZ	DPV	0.9 V	0.16 μM	0.5–200 μM	/	Sun et al., 2019a
MIP/GO@COF/GCE	Acetaminophen	DPV	0.4 V	0.032 μM	0.05–20 μM	/	Sun et al., 2019a
TAPB-DMTP-COFs/AuNPs/GCE	CGA	DPV	0.17 V	0.0095 μM	0.010–40 μM	/	Zhang et al., 2018a
COF_DHTA−TTA_/GCE	H_2_O_2_	DPV	−0.5 V −0.3 V	2.42 μM 1.70 μM	8.06–400 μM 5.66–400 μM	/	Xu M. et al., 2019
COF_DHTA−TTA_/GCE	pH	DPV	−0.5–0.5 V	/	11–3	64.2 mV/pH	Xu M. et al., 2019
GOD/COF_DHTA−TTA_/GCE	Glucose	DPV	−0.3 V −0.53 V	0.38 μM 0.18 μM	1.26–6,000 μM 0.60–6,000 μM	/	Xu M. et al., 2019
WP6@Ag@COF/GCE	PQ	CV	−0.755 V	0.014 μM	0.01–50 μM	/	Tan et al., 2019
TAPB-DMTP-COF/CPE	Lead	DPASV	−1.2 V	0.0019 μM	0.0050–2.0 μM	/	Zhang et al., 2018b
COF_ETTA−TPAL_-Fc (COOH)_2_/GCE	H_2_O_2_	DPV	−0.5/0.45 V	0.33 μM	1.1–500 μM		Liang et al., 2019b
MIP/MoS_2_/NH_2_-MWCNT@COF/GCE	SMR	DPV	1.03 V	0.11 μM	0.3–200 μM	/	Sun et al., 2019b
2HP6@Au@CP6@COF/GCE	SP	DPV	−0.4~-0.1 V	0.0017 μM	0.005–120 μM	/	Tan et al., 2020
COF_p−FeporNH2−BTA_/GCE	H_2_O_2_	DPV	−0.2 V	2.06 nM	6.85–7,000 nM	/	Xie et al., 2020a
COF_p−FeporNH2−BTA_/GCE	pH	DPV	−0.9–0.15 V	/	3–9	−41.2 mV/pH	Xie et al., 2020a
COF_Thi−TFPB_-CNT/GCE	pH	DPV	−0.6–0.3 V	/	1–12	54 mV/pH	Wang L. et al., 2020
COF_Thi−TFPB_-CNT/GCE	AA	DPV	−0.05 V	17.68 μM	53.04–4,000 μM/ 4–8 mM	/	Wang L. et al., 2020
CTpPa-2/GCE	BPA	DPV	0.2–1.0 V	0.02 μM	0.1–50 μM	/	Pang et al., 2020
CTpPa-2/GCE	BPS	DPV	0.2–1.0 V	0.09 μM	0.5–50 μM	/	Pang et al., 2020
TB-Au-COFs-Ab_2_/GCE	cTnI	SWV	−0.4 V	0.17 pg mL^−1^	0.5–10,000 pg mL^−1^	/	Zhang et al., 2018c
Fe_3_O_4_@AT-COF/MGCE	PNP	DPV	−0.772 V	0.2361 μM	10–3,000 μM	0.7588 μA cm^−2^μM^−1^	Wang Q. et al., 2020
Fe_3_O_4_@AT-COF/MGCE	ONP	DPV	−0.616 V	0.6568 μM	10–3,000 μM	0.7799 μA cm^−2^μM^−1^	Wang Q. et al., 2020
PtNPs@COFs-MWCNTs/GCE	Tanshinol	DPV	0.4 V	0.018 μM	0.002–1.1 mM	10.089 μA cm^−2^mM^−1^	Zhang et al., 2020
COF-3-BPPF_6_-CPE	HQ	DPV	0.17 V	0.31 μM	1–2,000 μM	/	Xin et al., 2020
COF-3-BPPF_6_-CPE	CC	DPV	0.26 V	0.46 μM	1–2,000 μM	/	Xin et al., 2020
GCE/DAT-COF	HQ	DPV	0.03 V	0.13 μM	0.20–500 μM	/	Arul et al., 2020
GCE/DAT-COF	CC	DPV	0.13 V	0.07 μM	0.20–500 μM	/	Arul et al., 2020
GCE/DAT-COF	RC	DPV	0.56 V	0.08 μM	0.20–500 μM	/	Arul et al., 2020
Fe_3_O_4_@NHCS/GCE	Dopamine	DPV	0.35 V	6.3 nM	0.01–40 μM	/	Lu et al., 2020
Fe_3_O_4_@NHCS/GCE	Uric acid	DPV	0.42 V	36.1 nM	0.10–40 μM	/	Lu et al., 2020
Fe_3_O_4_@NHCS/GCE	Guanine	DPV	0.75 V	143.2 nM	0.50–30 μM	/	Lu et al., 2020
Fe_3_O_4_@NHCS/GCE	Adenine	DPV	1.08 V	123.5 nM	0.50–40 μM	/	Lu et al., 2020
COF@NH_2_-CNT/GCE	Furazolidone	DPV	−0.4 V	77.5 nM	0.2–100 μM	/	Sun et al., 2020
Fe_3_O_4_@TAPB-DMTP-COFs/GCE	Luteolin	DPV	0.2 V	7.2 nM	0.010–7 μM	/	Xie et al., 2020b

### Application of COFs in Different Analytical Fields

#### Environmental Analysis

Endowed with an intrinsic absorption capability, COF-based electrochemical sensors have been widely used in the detection of environmental pollutants including hydrazine, explosives, catechol, nitrophenol, hydroquinone, bisphenol A, paraquat, and heavy metals. Porous and redox-active COFs were demonstrated to remove and detect hydrazine (Liang et al., 2020). Benefiting from a combination of the enhanced electron transfer and high surface area of DQ–COF, the electrochemical sensor exhibited a low detection limit, wide linear range, and high anti-interference ability. In addition, a sensitive and selective sensor was developed based on TAPB-DMTP-COFs for the detection of lead in an aqueous medium (Zhang et al., 2018b). This COFs were synthesized with 1,3,5-tris (4-aminophenyl) benzene (TAPB) and 2,5-dimethoxyterephaldehyde (DMTP). The novel sensor showed a broad linear range, low detection limit, high sensitivity, good stability and reproducibility, which may be assigned to the many active sites and high surface area of TAPB-DMTP-COFs. Under optimum conditions, the method showed an excellent linearity to the concentration of lead in the range of 0.0050–2.0 μM with a detection limit of 1.9 nM. This method not only demonstrates the feasibility of COF-based sensors for the detection of trace metal ions, but also broadens the detection range application of COF-based hybrid materials in electroanalytical chemistry. An Fe_3_O_4_-based magnetic COFs nanosphere (Fe_3_O_4_@AT-COFs) with a different surface morphologic structure is reported by facile ambient temperature synthesis, which shows the advantages of higher surface area, good water dispersity, long-term stability, excellent electrical conductivity, and pre-concentration effect (Wang Q. et al., 2020). The prepared Fe_3_O_4_@AT-COFs exhibited high electrocatalytic activity toward PNP and ONP, and the simultaneous detection of PNP and ONP was achieved with a wide linear detection range of 10–1,000 μM and low detection limits (LOD) of 0.2361 μM and 0.6568 μM, respectively.

#### Biomedical Analysis

In addition to its application in environmental pollution, an electrochemical sensing platform based on COFs also plays a prominent role in biomedicine, such as epidermal growth factor receptors, living cancer cells, prostate specific antigens, cardiac troponin I, glucose, ascorbic acid, dopamine, uric acid, guanine, adenine, luteolin, hydrogen peroxide, chlorogenic acid, furazolidone, tanshinol, sulfadiazine, and acetaminophen. Recently, porphyrin-based COFs (P-COFs) were synthesized, which are a potential candidate for the sensitive detection of target cancer markers or living cells (EGFR and living Michigan cancer foundation-7) (Yan et al., 2019). P-COFs presented high electrochemical activity, good stability in aqueous solution, excellent bio-affinity, and this material enabled strong immobilization of the aptamer strands. The fabricated aptasensor was demonstrated for the analysis of EGFR and living cancer cells, with the advantages of good anti-interferences ability, stability, and reproducibility.

An electroactive 2D COF_Thi−TFPB_ nanosheet packaged on amino-functionalized CNT was designed as a ratiometric electrochemical AA sensor, showing satisfactory selectivity, reproducibility, and stability (Wang L. et al., 2020). The COF_Thi−TFPB_ was synthesized by a dehydration condensation reaction between 1,3,5-tris (p-formylphenyl) benzene (TFPB) and thionine (Thi) and this porous crystalline material was a highly ordered 2D nanosheet. A highly selective and sensitive electrochemical sensing platform based on 2HP6@Au@CP6@COFs was successfully established for the determination of dangerous and explosive sodium picrate (SP) (Tan et al., 2020), in which Au nanoparticles play an electrocatalytic role and 2HP6 as well as CP6 contribute to the aggregation and identification of SP on the electrode surface. Recently, Xu described a facile one-pot strategy to immobilize COFs on an amino-functionalized carbon nanotube (NH_2_-CNT) support at room temperature via π-π interactions. The COFs-CNT composites modified electrode showed a high specific surface area (147.3 m^2^ g^−1^), and excellent electrical conductivity, which exhibited an excellent analytical performance for the detection of the nitrofuran antibacterial agent furazolidone (Sun et al., 2020).

### Application of Electroactive COFs in Electrochemical Sensors

**Figure 6:**
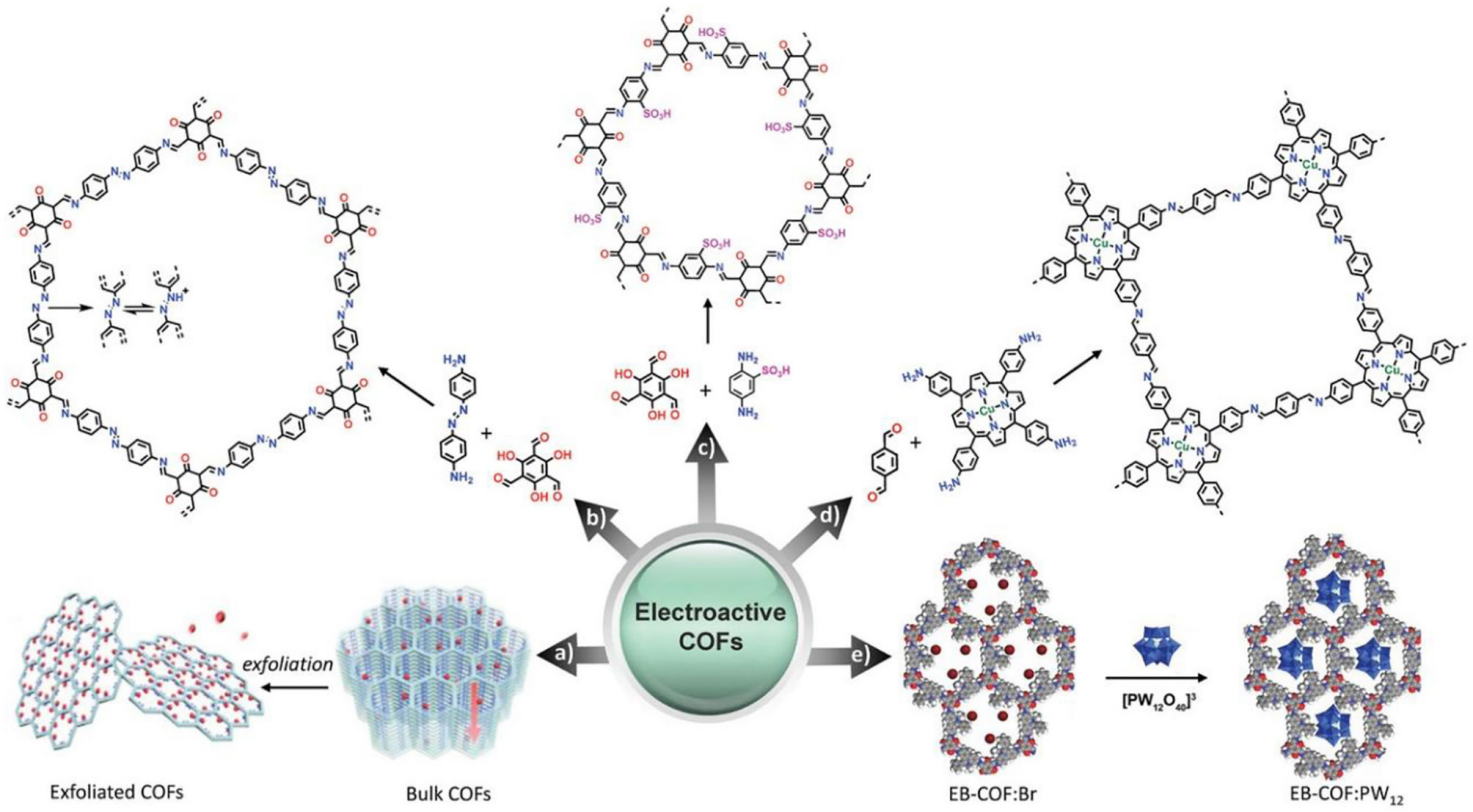
General design of electroactive COFs. (a) Typical design of electroactive bulk COFs and exfoliated COFs. (b) Typical bottom-up design of electroactive COFs with reactive skeletons. (c) Typical bottom-up design of electroactive COFs with reactive functional groups. (d) Typical bottom-up design of electroactive COFs with reactive metals. (e) Typical design of electroactive COF hybrids. (a) Reproduced with permission. Reproduced with permission from Yusran et al. (2020a). Copyright 2020, WILEY-VCH.

The incorporation of electroactive moieties in the structure endows electroactive COFs with great potential for electroanalysis application. Electroactive COFs possess abundant accessibly active sites which contribute to the electrochemical reaction and avoid overpotential. Electroactive COFs can be designed by incorporating electroactive sites (e.g., electron-rich species and metal) in their frameworks, or hybridizing COFs with other electroactive components with following scheme in Figure 6 (Yusran et al., 2020a).

An electroactive iron porphyrin-based covalent organic framework (COF_p−FeporNH2−BTA_) was synthesized via aldehyde-ammonia condensation reaction between 1,3,5-benzenetricarboxaldehyde and 5,10,15,20-tetrakis(4-aminophenyl)-21H, 23H-porphine, followed by post-modification with Fe^2+^. The synthesized COF*p*-Fepor NH2-BTA showed a good electrochemical redox property and electrocatalytic activity toward the reduction of hydrogen peroxide (Xie et al., 2020a). The electrochemical sensor based on COF_p−FeporNH2−BTA_ showed a wide linear range from 6.85 nM to 7 μM with the detection limit of 2.06 nM (S/N = 3) for the detection of hydrogen peroxide. Recently electroactive COFs were used to construct a ratiometric electrochemical sensor. Wang reported an electroactive COF with multiple redox-active states synthesized by an amine-aldehyde condensation reaction between 4, 4′,4″-(1,3,5-triazine-2,4,6-triyl) trianiline and 2,5-dihydroxy terethaldehyde (COF_DHTA−TTA_), which was applied for the construction of a ratiometric electrochemical sensor for the detection of hydrogen peroxide and pH level based on both current and potential signals (Xu M. et al., 2019). An electroactive COFs composite was also prepared by a dehydration condensation reaction between 1,3,5-tris(p-formylphenyl) benzene (TFPB) and thionine (Thi) wound with carbon nanotubes (CNT) to construct the ratiometric electrochemical sensing of ascorbic acid (Wang L. et al., 2020).

## Conclusions and Outlooks

This critical review briefly summarized the key properties of COFs that influence the electroanalytical performances, and corresponding solutions were discussed in detail, which will serve as a guide for the novel design and fabrication of an electrochemical sensor. Although COFs have been used to construct an electrochemical sensing platform for the sensitive analysis of biomedicine, environmental pollutants, and others, and some intriguing developments have been made, COFs in the electrochemical sensing field is still in its initial stage. To promote the development of electrochemical sensors based on COFs, the following challenges and outlooks should be considered in future work: (1) novel synthesis strategies (for example, microfluidic synthesis) need to be developed for conductive, nanoscale, and electroactive COFs for enhanced sensitivity and electrocatalytic activity. (2) Finding a simple and efficient surface modification method/strategy on conductive substrates is an urgent problem that needs to be resolved in the future. (3) The intrinsic fragility, powdered crystalline state, and large size of COFs lead to a low active area, low mass transfer rate, and difficult modification as well as poor stability on the electrode. Therefore, simple and facile synthesis methods for nanoscale and hydrogels/aerogels COFs need to be further investigated. (4) The antifouling capability and biocompatibility of COF-based materials still need to be studied and improved for the analysis of biological samples. (5) With the enhanced thermal, chemical, and mechanical stability, biomolecules encapsulated in COFs may be further explored to broaden their operational conditions and extend their potential applications in electrochemical sensors. (6) Molecularly imprinted COFs used in electrochemical sensors need to improve in their selectivity and sensitivity. (7) It will be an efficient method to improve the performances of conduction and sensing by using COFs as nanocarriers to encapsulate organic molecules that can be released through specific stimuli (Chang et al., 2019).

## Author Contributions

SC writing-original draft, table preparation, copyright application, and manuscript figure. BY writing-original draft, manuscript concept and design, funding acquisition, manuscript revision/review/editing, and manuscript figure. GL and DZ review/editing/revision. All authors contributed to the article and approved the submitted version.

## Conflict of Interest

The authors declare that the research was conducted in the absence of any commercial or financial relationships that could be construed as a potential conflict of interest.

## Abbreviations

2D: two-dimensional
3D: three-dimensional
2HP6: dihydroxylatopillar [6]arene
3D-KSC: 3D kenaf stem-derived macroporous carbon
AA: ascorbic acid
AAO: anodic aluminum oxide
AE: Au electrode
AP: amperometric
BDBA: 1,4-phenylenebis
BDT: 2,6-benzo[1,2-b:4,5-b′]dithiophene dialdehyde
BND: N-benzidine benzophenone imine
BPA: bisphenol A
BPPF_6_: N-butylpyridinium hexafluorophosphate
BPS: bisphenol S
BTA: 1,3,5-tricarbaldehyde
CC: catechol
CGA: chlorogenic acid
COF_p−porNH2−BTA_: iron-porphyrin-based covalent organic framework
COFs: covalent organic frameworks
CP6: cationic pillar [6]arene
CTnI: cardiac troponin I
CV: cyclic voltammetry
DAAQ: 2,6-diaminoanthraquinone
DAB: p-phenylenediamine
DAT: 3,5-diamino-1,2,4-triazole
DHTA: 2,5-dihydroxyterethaldehyde
DMTP: 2,5-dimethoxyterephaldehyde
DPASV: differential pulse anodic stripping voltammetry
DPPD: 3,8-diamino-6-phenylphenanthridine
DPV: differential pulse voltammetry
EGFR: epidermal growth factor receptor
EIS: electrochemical impedance spectroscopy
ETTA: 4,4′,4″,4‴-(1,1,2,2-ethylenetetrayl)tetrakisaniline
Fe_3_O_4_@NHCS: Fe_3_O_4_/N co-doped hollow carbon spheres
FTO: fluorine doped tin oxide
GCE: glassy carbon electrode
GO: graphene oxide
GOD: glucose oxidase
HQ: hydroquinone
ITO: indium tin oxide
LOD: low detection limits
MCOFs: metal-covalent organic frameworks
MGCE: magnetic glassy carbon electrode
MIP: molecularly imprinted polymer
MOFs: metal-organic frameworks
NH_2_-f-MWCNT: amino-functionalized carbon nanotube
NPs: nanoparticles
ONP: o-nitrophenol
ORR: oxygen reduction reaction
P-COFs: porphyrin-based covalent organic frameworks
PDA: 1,4-phthalaldehyde
PEDOT: poly(3,4-ethylenedioxythiophene)
PNP: p-nitrophenol
POR: poly(5,10,15,20-tetrakis (4-aminophenyl)porphyrin)
PQ: paraquat
PSA: prostate specific antigen
PSF: polysulfone
PVP: polyvinylpyrrolidone
RC: resorcinol
SDZ: sulfadiazine
SMR: sulfamerazine
SP: sodium picrate
SWV: square wave voltammetry
TAPB: 1,3,5-tris (4-aminophenyl) benzene
TAPP: 5,10,15,20-tetrakis(4- aminophenyl)porphyrin
TCNQ: tetracyanoquinodimethane
TFB: 1,3,5-triformylbenzene
TFP: 1,3,5-triformylphluroglucinol
TFPB: 1,3,5-tris(p-formylphenyl) benzene
Thi: thionine
TPAL: terephthalaldehyde
TpBD: 1,3,5-triformylphloroglucinol (Tp)/benzidine (BD)
TTA: 4,4′,4″-(1,3,5-triazine-2,4,6-triyl)trianiline
TTF: tetrathiafulvalene
WP6: water-soluble pillar[6]arene.
